# Motoneuron persistent inward current contribution to increased torque responses to wide-pulse high-frequency neuromuscular electrical stimulation

**DOI:** 10.1007/s00421-024-05538-8

**Published:** 2024-06-28

**Authors:** Timothée Popesco, Lucas Bet da Rosa Orssatto, François Hug, Anthony John Blazevich, Gabriel Siqueira Trajano, Nicolas Place

**Affiliations:** 1 Institute of Sport Sciences, University of Lausanne, Lausanne, Switzerland; 2 Faculty of Health, School of Exercise and Nutrition Sciences, Institute for Physical Activity and Nutrition (IPAN), Deakin University, Geelong, Australia; 3 LAMHESS, Université Côte d’Azur, Nice, France; 4 School of Biomedical Sciences, The University of Queensland, Brisbane, QLD Australia; 5 School of Medical and Health Sciences, Centre for Human Performance, Edith Cowan University, Joondalup, WA Australia; 6 Faculty of Health, School of Exercise and Nutrition Sciences, Queensland University of Technology (QUT), Brisbane, Australia

**Keywords:** Motor unit, ∆*F*, NMES, PIC, Plantar flexion

## Abstract

**Purpose:**

To assess the effect of a remote handgrip contraction during wide-pulse high-frequency (WPHF) neuromuscular electrical stimulation (NMES) on the magnitude of extra torque, progressive increase in torque during stimulation, and estimates of the persistent inward current (PIC) contribution to motoneuron firing in the plantar flexors.

**Methods:**

Ten participants performed triangular shaped contractions to 20% of maximal plantar flexion torque before and after WPHF NMES with and without a handgrip contraction, and control conditions. Extra torque, the relative difference between the initial and final torque during stimulation, and sustained electromyographic (EMG) activity were assessed. High-density EMG was recorded during triangular shaped contractions to calculate ∆*F*, an estimate of PIC contribution to motoneuron firing, and its variation before vs after the intervention referred to as ∆*F* change score.

**Results:**

While extra torque was not significantly increased with remote contraction (WPHF + remote) vs WPHF (+ 37 ± 63%, *p* = 0.112), sustained EMG activity was higher in this condition than WPHF (+ 3.9 ± 4.3% MVC EMG, *p* = 0.017). Moreover, ∆*F* was greater (+ 0.35 ± 0.30 Hz) with WPHF + remote than control (+ 0.03 ± 0.1 Hz, *p* = 0.028). A positive correlation was found between ∆*F* change score and extra torque in the WPHF + remote (*r* = 0.862, *p* = 0.006).

**Discussion:**

The findings suggest that the addition of remote muscle contraction to WPHF NMES enhances the central contribution to torque production, which may be related to an increased PIC contribution to motoneuron firing. Gaining a better understanding of these mechanisms should enable NMES intervention optimization in clinical and rehabilitation settings, improving neuromuscular function in clinical populations.

**Supplementary Information:**

The online version contains supplementary material available at 10.1007/s00421-024-05538-8.

## Introduction

Neuromuscular electrical stimulation (NMES) is widely used as a tool to improve or restore neuromuscular function in a variety of health conditions (Maffiuletti et al. 2018). Wide-pulse high-frequency (WPHF) NMES (usually 1-ms pulses delivered at 100 Hz) has been developed to overcome some of the limitations associated with conventional NMES (pulse duration: 0.1–0.5 ms, stimulation frequency: 50–100 Hz) (Bergquist et al. 2011; Vanderthommen and Duchateau 2007). Indeed, the evoked torque can be higher in response to WPHF NMES than conventional NMES, due to a progressive increase in torque production during the stimulation referred to as ‘extra torque’ (Bergquist et al. 2011; Collins 2007), although it does not occur in all individuals (Wegrzyk et al. 2015a; Regina Dias Da Silva et al. 2015; Neyroud et al. 2018). For example, tetanic contractions induced by WPHF NMES usually require a lower stimulation intensity to achieve a given torque output (usually ~ 10% maximal voluntary contraction (MVC) torque), reducing discomfort when compared to conventional NMES parameters. Even though maximal evocable torque has been shown to be slightly higher with WPHF as compared to conventional NMES (Espeit et al. 2023), extra torque is expected to be higher at submaximal stimulation intensity. In fact, wide pulses favor activation of the large diameter sensory axons as they have a longer strength-duration time constant and a lower rheobase compared with the terminal axonal branches of motoneurons (Lagerquist and Collins 2008). This in turn elicits contractions with a contribution from the reflexive Ia-pathway, which should result in the recruitment of lower threshold motor units, allowing a more physiological recruitment pattern (Collins et al. 2002) than the non-selective, random recruitment pattern usually described under conventional NMES (Bickel et al. 2011).

One of the main hypotheses to explain the extra torque production is the amplification of motor neuron output due to the presence of persistent inward currents (PICs) within the motoneurons themselves (Bergquist et al. 2011; Collins 2007; Donnelly et al. 2021; Neyroud et al. 2018). PICs are depolarizing calcium and sodium currents that amplify and prolong motoneuron output for a given synaptic input (Heckman and Enoka 2012). The high-frequency repetitive stimulation of Ia afferents, preferentially activated by trains of longer NMES pulses, is thought to initiate PICs in motoneuron dendrites (Dideriksen et al. 2015). Consequently, motoneuron discharge rates are increased, thus increasing the force output of the motor units for a given NMES intensity. Although the presence of sustained electromyographic (EMG) activity (i.e., EMG activity persisting after the end of the stimulation) can be used to indirectly highlight the presence of PICs in response to WPHF NMES (Donnelly et al. 2021; Neyroud et al. 2018; Trajano et al. 2014), PICs contribution to motor neuron firing can be estimated in humans using the paired motor unit technique (Gorassini et al. 2002) which involves measuring of motor unit spiking activity with either indwelling EMG electrodes or surface, high-density (HD) EMG electrodes. With this technique, pairs of motor units recruited during triangular shaped contractions are used to quantify discharge rate hysteresis, a prominent functional outcome of motoneuronal PIC activation. To do this, the difference in discharge rates of a lower threshold ‘control’ unit at the instants of recruitment and derecruitment of a higher threshold ‘test’ unit (delta frequency, Δ*F*) is computed. Δ*F* is suggested to represent the contribution of PICs to motoneuron firing and has been demonstrated to be sensitive to change after acute interventions (Mackay Phillips et al. 2023; Mesquita et al. 2021; Orssatto et al. 2022; Udina et al. 2010).

The level of monoaminergic input onto the spinal cord influences PIC amplitude (Lee and Heckman 2000). For instance, serotonin has been suggested to be an important neuromodulator of PIC-related motoneuronal gain (Heckman et al. 2008; Wei et al. 2014). Two recent studies (Mackay Phillips et al. 2023; Orssatto et al. 2022) observed an increase in ∆*F* of lower limb muscles after contraction of a muscle distant to the examination site (handgrip contraction), known as a remote contraction (Masugi et al. 2019). As serotonergic projections to the spinal cord are diffuse (Aghajanian and Liu 2009), an increase in the motor activity of one muscle group should increase the PIC-related gain of motoneurons in other, remote muscle groups (Wei et al. 2014). Therefore, if PICs are effectively involved in the extra torque produced by WPHF NMES, a simultaneous remote contraction should increase extra torque, and be accompanied by an increased ∆*F* and sustained EMG activity. The purpose of the present study was to assess the effect of a remote (handgrip) contraction during WPHF NMES of the plantar flexors on the magnitude of extra torque and estimates of PIC amplitudes. We hypothesized that the handgrip contraction would increase WPHF NMES-derived extra torque production concomitantly with an increase in Δ*F*. The identification of an intervention amplifying the exerted extra torque would improve the efficiency of the use of WPHF NMES as a (re)training tool. The mechanisms behind extra torque production remain unclear and this is the first study aiming at linking extra torque with PIC estimates from HD EMG measurements, which motivated its exploratory design.

## Materials and methods

### Participants

Ten participants (1 woman and 9 men, 35 ± 7 years, 176 ± 8 cm, 81 ± 13 kg) without any neuromuscular disorder volunteered to participate in this study. Participants were instructed to avoid vigorous exercise and alcohol consumption for 24 h, and caffeine use for at least 6 h, prior to testing. All participants read and signed the informed consent document, and the QUT’s University Human Research Ethics Committee approved this study (1800000550).

### Experimental protocol

**Fig. 1:**
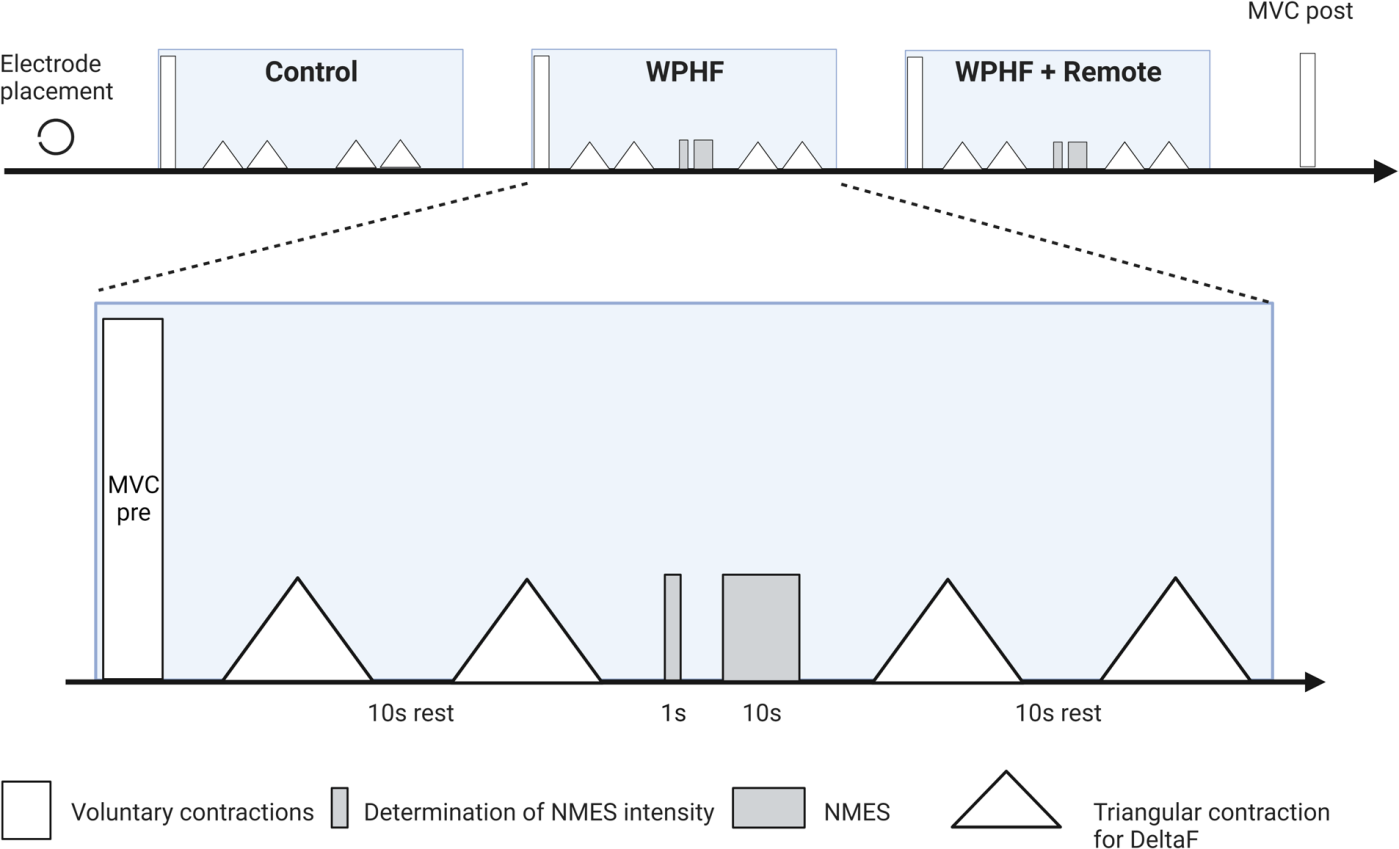
Protocol outline. The three conditions were separated by 5 min of rest and performed in a random order. *MVC* maximal voluntary contraction, *WPHF* wide-pulse high-frequency neuromuscular electrical stimulation, *NMES* neuromuscular electrical stimulation

All participants were involved in other studies using the same techniques in the last six months and therefore no familiarization session was needed. The experiments were performed on the right limb and involved three conditions separated by 5 min of rest and completed in random order: (a) resting period of 40 s (control), (b) 20-s train of WPHF NMES (100 Hz, 1 ms) at the intensity required to evoke an initial torque corresponding to 10% of MVC torque, and (c) a WPHF + Remote condition, similar to *b* but with an ipsilateral handgrip contraction during the 20-s tetanic contraction (Fig. 1). The intensity and duration of stimulation trains were chosen in accordance with previous WPHF NMES studies (Wegrzyk et al. 2017; Wegrzyk et al. 2015a, b) In each condition, triangular shaped contractions were performed before and after the intervention to assess PICs. Before testing, 10 to 12 voluntary isometric plantar flexor warm-up contractions ranging from 20 to 80% of the estimated maximum torque were executed and held for 2–3 s before the participant performed 2–3 MVCs until two trials exhibited a difference of < 5% (with rest intervals of 60 s between trials). The participants were instructed to generate their maximum torque within 1–2 s and sustain it for approximately 3 s. In the resting control condition, participants were instructed to refrain from voluntarily contracting any muscles for 40 s. In WPHF, 20-s of WPHF stimulation was applied to the plantar flexors while the participant remained as relaxed as possible (see details below). In WPHF + Remote, participants commenced the handgrip contraction by squeezing a 6.6-cm diameter rubber ball approximately 3 s before the start of the 20-s WPHF stimulation train, to maintain the handgrip contraction at ~ 40% of estimated maximal handgrip torque (Orssatto et al. 2022), and then to stop the contraction after the stimulation was ceased. In all conditions, the participants performed two voluntary, triangular-shape ramped contractions from 0 to 20% MVC and then back to 0% with 10-s ramp up and 10-s ramp down durations (2%MVC/s rate of torque change) before and immediately after the 40-s period with visual feedback of their torque and the required target torque. The two contractions were separated by 10 s of rest. The participants were asked to perform a third triangular shaped contraction if neither of the first two contractions followed the requested torque-time trajectory with sufficient precision, assessed as an absence of visible increase or drop of the torque of more than 5% MVC torque within 0.5 s and less than 2% MVC torque difference between the realized and the expected path during the whole contraction period.

### Data collection and analysis

#### Electrical stimulation

NMES was delivered transcutaneously by a constant current stimulator (Digitimer, DS7AH, Hertfordshire, UK) using two active electrodes. The anode (5 cm × 10 cm, Uni-Patch R series, Covidien, Dublin, Ireland) was placed on the widest part of the gastrocnemius muscles, (~ 4 cm below the popliteal fossa) and the cathode (5 cm × 10 cm, Uni-Patch R series, Covidien, Dublin, Ireland) was placed on the soleus below the gastrocnemii (Donnelly et al. 2021). The stimulator was triggered by the LabChart software (LabChart 8, AD Instruments, Sydney, Australia). The WPHF NMES stimulation intensity was determined using short 1-s, 100-Hz tetanic contractions to reach 10% MVC torque and therefore limit any influence of extra torque.

#### Torque

Torque data were collected using a Biodex system 4 (Biodex Medical Systems, New York, US) connected to a computer running the LabChart software (LabChart 8, AD Instruments, Sydney, Australia) at a 1000 Hz analog–digital conversion rate. Participants were seated on the Biodex chair with hip flexed ~ 70° (0° = full extension), knee flexed at 90° (0° = full extension), and ankle at anatomic position. The participants’ feet were strapped across the dorsum of the foot at the metatarsals. The peak torque obtained in the MVCs at the start of each session was considered the MVC torque.

As in previous studies (Collins et al. 2001; Wegrzyk et al. 2015b), the extra torque during stimulations was calculated as the percentage of the torque difference between the last second (final torque) and the 2nd second of stimulation (initial torque): extra torque = (final torque − initial torque) / initial torque) × 100.

#### Bipolar surface electromyography

Soleus EMG signals were recorded using circular self-adhesive silver chloride (Ag/AgCl) electrodes. The electrodes (1-cm diameter, Meditrace 100, Tyco, Markham, Canada) were positioned lengthwise over the soleus muscle belly at two-thirds of the distance between the medial condyle of the femur and the medial malleolus with a 2-cm inter-electrode distance, according to SENIAM recommendations (Hermens et al. 2000). Soleus myoelectrical activity during conditions was estimated using the root mean square (RMS) of the EMG signal. During MVCs, the mean value within a 500-ms interval surrounding the peak torque (250 ms before and 250 ms after) was obtained. This value was used to normalize the sustained EMG activity, referred as the mean EMG amplitude taken during the 500-ms window after the WPHF stimulus period. This 500-ms window was taken 125 ms (integration time for the RMS calculation of the EMG signal) after the last stimulation artifact to avoid any contamination in the measurement of the sustained EMG activity. Furthermore, in cases where the EMG activity was persisting for more than 15 s, participants were asked to perform a dorsiflexion to stop sustained EMG activity/sustained torque.

#### High-density electromyography

High-density EMG was also recorded from soleus during contractions using one semi-disposable 32-channel electrode grid with 10-mm inter-electrode distances (ELSCH032NM6, OTBioellettronica, Torino, Italy) placed over the medial side of the *soleus* muscle (see online resource 1) just below the stimulation electrode and directed toward the calcaneus tendon (Orssatto et al. 2021). A strap electrode was dampened and positioned around the ankle joint as a ground electrode. EMG signals were recorded in monopolar mode, amplified (256×), then sampled at 2000 Hz using a 16-bit wireless amplifier unit (Sessantaquattro, OTBioelletronica) before being bandpass filtered (10–500 Hz).

For all HD-EMG analyses, the first triangular shaped contraction was preferentially used; the following contraction was used if the first or second contractions was performed with insufficient precision (see *Experimental protocol*). The EMG signals were decomposed into single motor unit discharge events using a convolutive blind source separation algorithm, the convolutive kernel compensation (CKC) method, using the DEMUSE software (Holobar and Zazula 2007). Motor unit filters, which define the influence of each motor unit on the combination of EMG channels in both time and space and thus allow the estimation of the individual motor unit spike train, were used to track the same motor unit within each condition by applying the CKC method on concatenated recordings obtained before and after the intervention (Frančič and Holobar 2021, 2022). After automatic decomposition, all motor unit spike trains were visually inspected and manually edited to correct for false positive and false negative firings (Del Vecchio et al. 2020; Hug et al. 2021; Orssatto et al. 2021). Only motor units with a pulse-to-noise ratio above 30 dB were retained for further analysis. Subsequently, discharge events with interspike intervals below 0.025 s and above 0.4 s were excluded, and discharge events were converted into instantaneous discharge rates calculated in pulses per second and fitted with a 5th order polynomial function (Orssatto et al. 2021; Trajano et al. 2014). Then, the discharge rate of a low-threshold motor unit at the time of recruitment and de-recruitment of a higher threshold motor unit was assessed as this provides an indication of the level of synaptic drive received by the higher threshold unit. The difference in instantaneous discharge rate between the time of recruitment and de-recruitment of the higher threshold unit is referred to as ∆*F* and is considered to be proportional to PIC strength (Powers et al. 2008). ∆*F*s were calculated for pairs of motor units with a rate-rate correlation between the smoothed discharge rate polynomials equal to or above 0.7, with test unit recruited at least 0.5 s after the control unit, and when no saturation of discharge rates was detected on the control unit (discharge rate increased by at least 0.5 Hz after the recruitment of the test unit) (Stephenson and Maluf 2011). ∆*F* values were averaged for each participant to allow testing for potential correlations with WPHF NMES parameters. The absolute difference between ∆*F*s after versus before the intervention in each condition is referred to as the ∆*F* change score.

### Statistics

Statistics were performed using Jamovi software (version 2.2.5.0, Sydney, Australia) and data were plotted in graphical format using GraphPad Prism (GraphPad Software 8, Inc., San Diego, CA, USA). Verification of normality was performed with the Shapiro–Wilk test. Student paired t tests were used to compare extra torque and sustained EMG activity between WPHF and WPHF + remote conditions. Cohen’s d was used to calculate effect size and was interpreted following Cohen’s guidelines with values over 0.8 considered as a large effect, over 0.5 as a moderate effect and over 0.2 considered as a small effect (Cohen 1988). ANOVAs were used to test for between-condition differences in MVC torque, peak discharge rate and recruitment thresholds after distribution normality was verified. Friedman’s ANOVAs were used to compare ∆*F* change score between conditions and Wilcoxon’s rank test were used to compare ∆*F* within conditions as the data were not normally distributed. Spearman correlation coefficients were used to quantify the strength of the linear relationship between ∆*F* and both extra torque and sustained EMG activity. Data are presented as mean ± SD in the text and individual values in the figures. Statistical significance was set at an alpha level of *p* < 0.05.

## Results

**Fig. 2:**
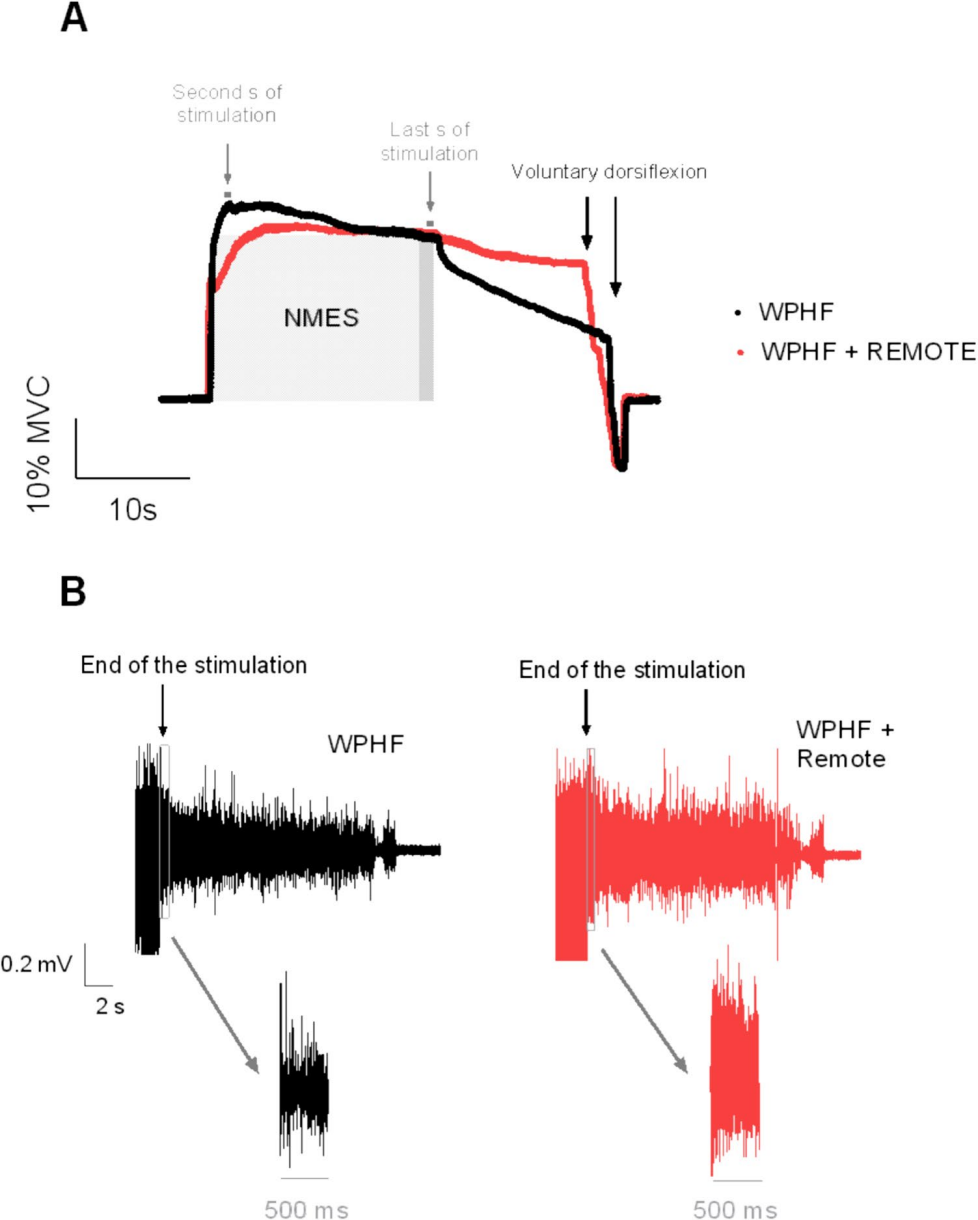
Original torque traces (**A**) and sustained EMG activity (**B**) from a representative participant in WPHF and WPHF + remote. The second s and last s of stimulation for the measurement of extra torque as well as the window of 500 ms considered for sustained EMG activity quantification are displayed. Positive values represent plantar flexion while negative values account for dorsiflexion. A voluntary ankle dorsiflexion was performed by the participant 15 s after the end of the stimulation and successfully stopped the sustained torque

Raw torque and EMG data captured during a WPHF NMES stimulation train both with and without handgrip contraction are shown in Fig. 2.

**Fig. 3:**
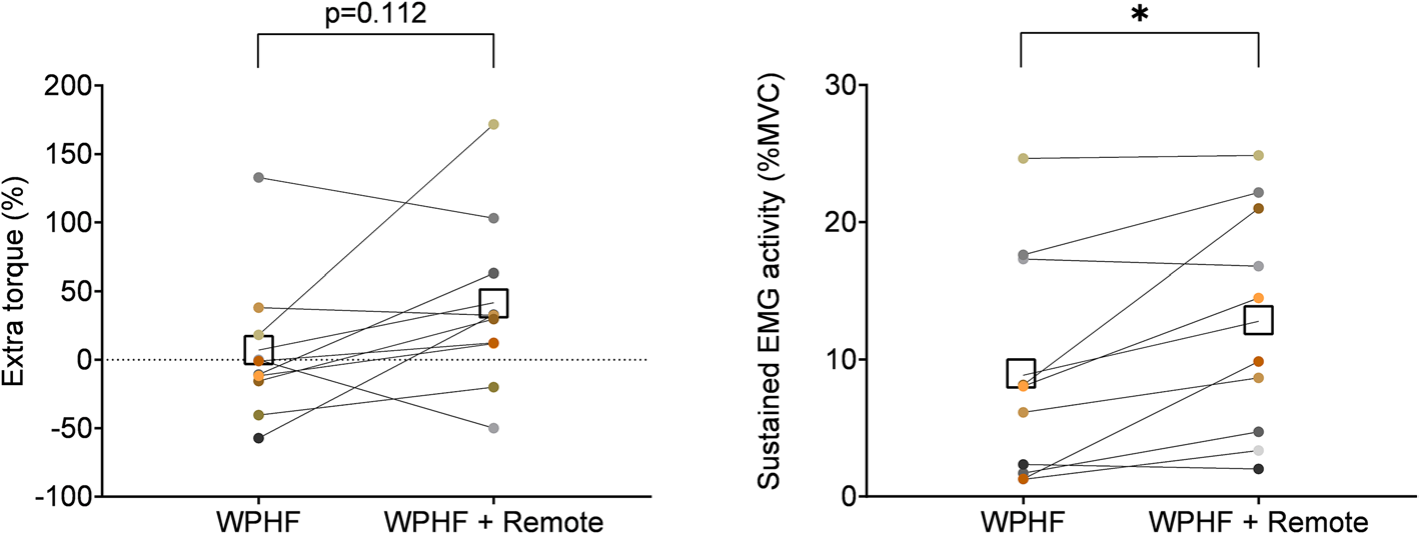
Extra torque (**A**) and sustained EMG activity (**B**) in WPHF and WPHF + remote. Individual values are displayed, and the means are presented as squares. *Significant difference at *p* < 0.05

At the group level, extra torque was not statistically different in WPHF + remote compared to WPHF (respectively 42 ± 66% vs 7 ± 55%, *p* = 0.112, Cohen’s *d* = 0.56) although a large variability in the response was observed across participants (Fig. 3A). However, sustained EMG activity was significantly higher in WPHF + remote than WPHF (respectively 12.8 ± 9.3% EMG_MVC_ vs 8.8 ± 8.8% EMG_MVC_, *p* = 0.017, Cohen’s *d* = 0.92) (Fig. 3B).

**Fig. 4:**
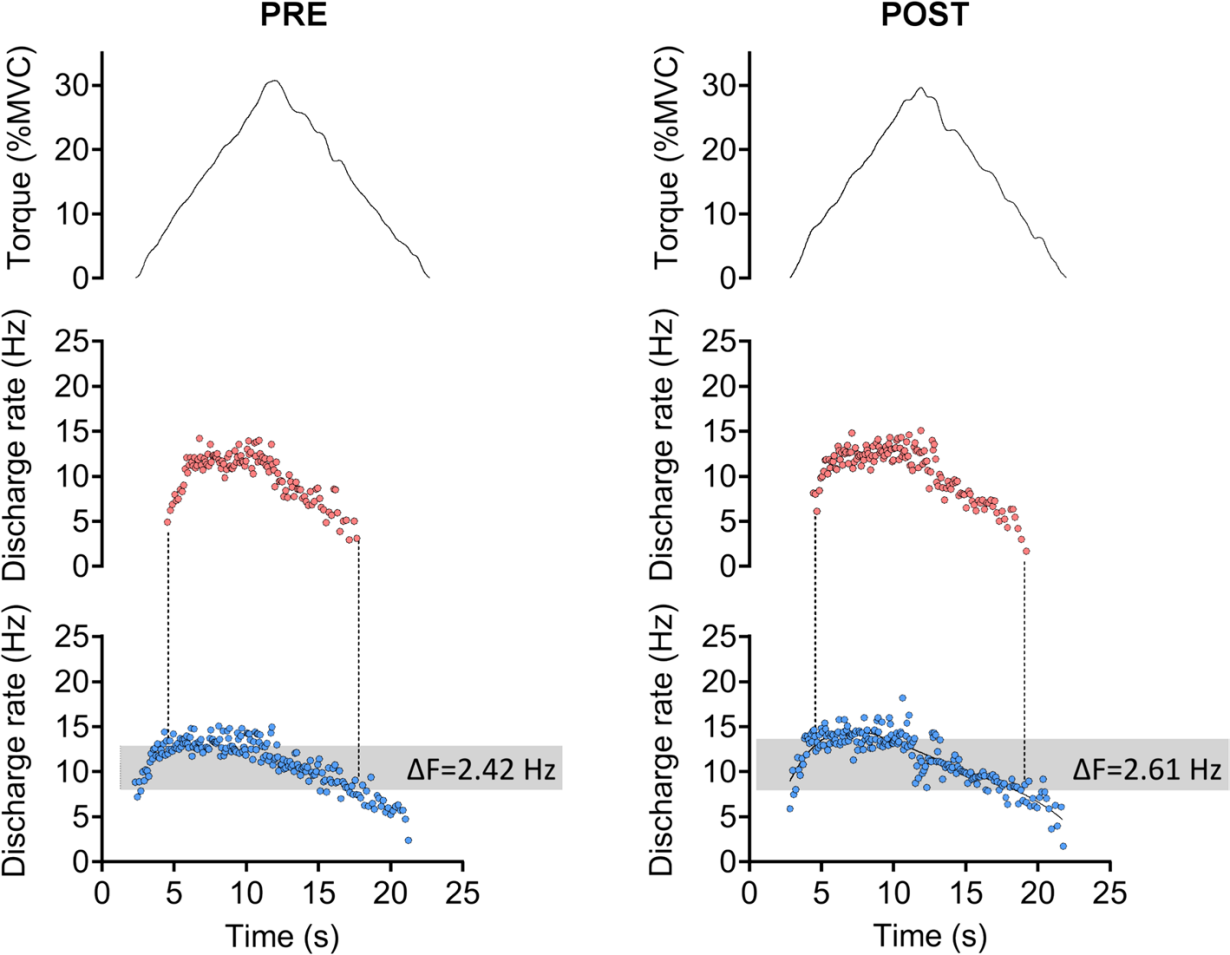
Original torque traces and discharge rates of a test motor unit (red) and a control motor unit (blue) from a representative participant in WPHF + remote

A total of 44, 49 and 47 motor units were identified among 8 of the 10 participants respectively in control, WPHF and WPHF + remote with a mean of 5.8 ± 2.1 motor units for the included participants (see online resource 2). In these conditions, 28, 26 and 26 motor units were respectively tracked before and after the intervention allowing to assess 80 ∆*F* values on pairs of motor units following the criteria for ∆*F* assessment (see Fig. 4 for original recordings). In WPHF + remote, ∆*F* was higher after than before stimulation (*p* = 0.016) (Table 1) and the difference between pre- and post-intervention was not significant in the other conditions (*p* = 0.461 in control and *p* = 0.383 in WPHF, Table 1). Motor unit recruitment thresholds were not significantly different between conditions (*p* = 0.507) but were significantly lower in post- than pre-intervention for WPHF + remote (*p* = 0.040, Table 1).

**Table 1:** Mean ± SD for ∆*F*, peak discharge rate, and recruitment threshold for control, WPHF and WPHF + remote

	∆*F* (Hz)	Peak discharge rate (Hz)	Recruitment threshold (%MVC)
*Pre-intervention*			
Control	2.33 ± 0.82	9.95 ± 0.90	7.2 ± 4.4
WPHF	2.15 ± 0.93	10.40 ± 1.25	7.7 ± 4.8
WPHF + remote	2.23 ± 0.91	10.40 ± 1.19	7.7 ± 4.6
*Post-intervention*			
Control	2.36 ± 0.76	10.20 ± 0.95	7.2 ± 4.5
WPHF	2.30 ± 0.91	9.90 ± 1.42	7.8 ± 5.0
WPHF + remote	2.59 ± 1.05	10.50 ± 1.54	7.2 ± 4.5
*Post–pre difference*			
Control	0.03 ± 0.10	0.28 ± 0.34	− 0.1 ± 2.7
WPHF	0.15 ± 0.32	− 0.49 ± 0.54	0.1 ± 1.8
WPHF + remote	0.35 ± 0.30^†^	0.15 ± 0.55^‡^	− 0.5 ± 1.4*

^†^Different from control, *p* < 0.05; ^‡^different from WPHF, *p* < 0.05, *post–pre difference within a condition

**Fig. 5:**
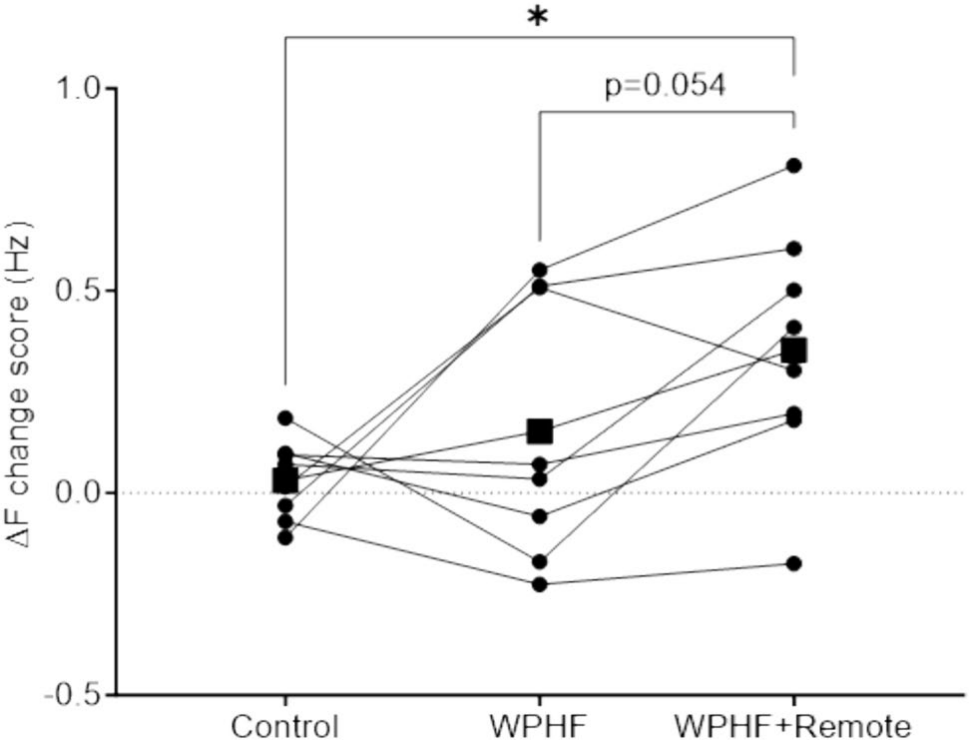
∆*F* change score. Individual values are displayed, and means are presented as squares. *Significant difference at *p* < 0.05

The ∆*F* change score was significantly different between conditions (*p* = 0.042). Post hoc tests revealed that the variation in ∆*F* with WPHF + remote was higher compared to control (*p* = 0.028) and close to significance when compared to WPHF alone (*p* = 0.054) (Fig. 5).

**Fig. 6:**
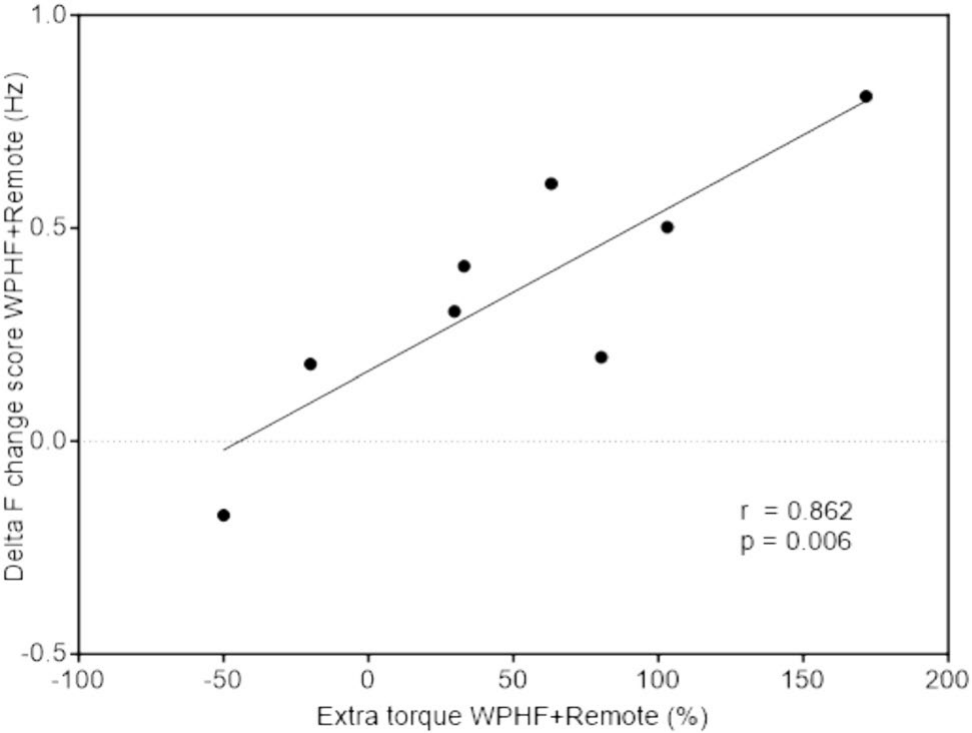
Correlation between ∆*F* change score and extra torque in the WPHF + remote condition

The change in ∆*F* in WPHF + remote was positively and strongly correlated with the magnitude of extra torque (Fig. 6) but not with the sustained EMG amplitude (*r* = 0.113, *p* = 0.791). The change in peak discharge rate was significantly different between conditions (*p* = 0.011), significantly higher in WPHF + remote compared to WPHF (*p* = 0.046). No correlation was observed between the change in ∆*F* and extra torque production (*r* = − 0.008, *p* = 0.984) or sustained EMG activity in WPHF (*r* = 0.175, *p* = 0.679).

MVC torque measured before each condition remained unchanged (control: 154 ± 44, WPHF: 151 ± 41, WPHF + remote: 151 ± 44 N m, *p* = 0.879).

## Discussion

The purpose of this study was to assess the effect of a remote contraction during WPHF NMES of the plantar flexors on the magnitude of extra torque, sustained EMG, and ∆*F*. The remote contraction was used as a way to increase serotoninergic input onto the motoneurons. We hypothesized that the remote contraction would elicit an increase in extra torque production during WPHF due to higher PIC contribution to motoneuron firing (i.e., ∆*F*). The results showed that ∆*F* was increased after WPHF + remote and that in this condition, the increase in ∆*F* was correlated with the extra torque. We also observed that the sustained EMG activity in WPHF + remote was higher than in the WPHF condition.

The extra torque generated in response to WPHF NMES was generally higher when the stimulation was combined with a voluntary handgrip contraction than the stimulation alone, although this effect did not reach statistical significance. Even though the effect size can be interpreted as moderate (Cohen 1988), the absence of significance most likely results from the small sample size and high interindividual variability (see Fig. 3A) with four of the ten participants exhibiting extra torque, in line with previously reported observations (Donnelly et al. 2021; Neyroud et al. 2014). However, the combination of NMES with a remote contraction seems to enhance the reflexive contribution to torque production as it is accompanied by an increase in the sustained EMG activity with a large effect size (Cohen 1988). The remote contraction was able to generate extra torque in response to WPHF NMES on three participants who did not exhibit any in the WPHF condition (Fig. 3); this change in status from non-responder to responder had already been observed in a previous study after three weeks of WPHF training (Neyroud et al. 2019). Moreover, ∆*F* was increased after WPHF + remote and the increase in peak discharge rate was higher during WPHF + remote than WPHF alone. As there was no significant effect found when comparing the WPHF condition to the control condition, our findings suggest that the effect of WPHF NMES on PIC strength is amplified by the remote contraction and this results from the cumulative effects of WPHF and the remote contraction. These results confirm previous findings that handgrip contraction can increase estimates of PICs and motor unit discharge rates in the plantar flexors (Mackay Phillips et al. 2023; Orssatto et al. 2022) and are the first to show that handgrip contraction can also enhance the sustained myoelectrical activity in response to WPHF NMES. Normal motor behavior relies significantly on the presence of serotonin, which modulates PIC-related motoneuron input–output gain (Wei et al. 2014). Serotonin levels increase proportionally with the demand for higher levels of force output (Veasey et al. 1995), increasing the contribution of PICs to motoneuron firing output (Heckman et al. 2009; Orssatto et al. 2021). It has been suggested that the increase in PICs in *soleus* due to a handgrip contraction relies on monoaminergic drive from the brainstem nuclei (Mackay Phillips et al. 2023). The diffuse and unorganized descending projections of the monoaminergic system in the central nervous system induce a diffuse control of spinal motor excitability (Heckman et al. 2008). Through non-synaptic communication, serotonin can diffuse widely and increase excitation in muscle groups far from the intervention site, which can facilitate motor unit recruitment. It is thought to be the main mechanism for PIC facilitation (Orssatto et al. 2022). Lower recruitment thresholds were observed after WPHF + remote than beforehand for the same motor units (Table 1). It can be interpreted as further evidence of the excitatory effect of the NMES plus remote contraction combination.

PICs have a broad impact on synaptic integration and can substantially increase motoneuron excitability (Heckman et al. 2008), which can amplify reflexive responses and induce spontaneous motoneuron activity (Bergquist et al. 2011; Collins et al. 2001). The effects of PICs on motoneuron firing, including synaptic input amplification, self-sustained firing, and facilitation or warm-up effects have been suggested to play a role in extra torque production (Binder et al. 2020; Collins et al. 2001). While ∆*F* has been validated as an effective method to estimate PIC amplitude (Gorassini et al. 2002; Powers and Heckman 2015), sustained EMG activity has also recently been suggested to be an indicator of the presence of PICs (Donnelly et al. 2021). In this study, the concomitant increase in ∆*F* and sustained EMG activity (Figs. 3B, 5) suggests that PICs might contribute to extra torque production. Indeed, there are strong similarities between amplification and prolongation of the motor unit discharge rate when PICs are activated (Heckman and Enoka 2012) and torque production during WPHF NMES, which may progressively increase during stimulation (extra torque) and can persist after cessation of the stimulation.

The correlation between the change in ∆*F* and the extra torque generated during stimulation in WPHF + remote supports the possible implication of PICs in the extra torque production, suggesting that PIC facilitation, which is estimated by the ∆*F* value and supported by the increased peak discharge rate in this condition, may contribute to the production of extra torque. Recently, responses to NMES used concomitantly with tendon vibration have been suggested to be an indirect estimation of PIC behavior as a warm-up effect, i.e., the progressive increase in torque in response to the combined use of vibration and NMES, was observed (Mesquita et al. 2021; Trajano et al. 2014). Tendon vibration induces repetitive and slight changes in muscle length, which stimulates muscle spindles and causes an excitatory response from Ia afferents, reflexively activating motor units (Grande and Cafarelli 2003). We contend that comparable neurophysiological mechanisms are involved for extra torque generation in response to WPHF NMES and our results highlight this expectation as PIC estimates and extra torque showed a comparable increase. However, the absence of correlation between sustained EMG activity and ∆*F* in our results suggests that the mechanisms underlying the modulation of ∆*F* and sustained EMG activity may differ, i.e., that these two variables reflect different properties of PICs. We thus propose that the sustained EMG activity might be an indicator of the presence of PICs (Donnelly et al. 2021), but its use to estimate the amplitude of PICs is more nuanced.

The present results highlight underlying mechanisms of NMES-induced muscle contractions, which are important to understand in order to ultimately optimize the effectiveness of NMES interventions in clinical and rehabilitation settings. Here, the design of the study was exploratory (ten participants) and set the basis for future works in the area, including other strategies to enhance extra torque in response to WPHF NMES. In conclusion, the present study showed that ∆*F* is increased after a WPHF NMES-evoked contraction combined with a remote contraction and that this increase may be responsible for the higher central contribution to the NMES response. Considering the significant impact of serotonin and noradrenaline on PICs, it may be of interest to explore the option of modifying their concentrations in the spinal cord using various interventions that modulate monoamine concentration. Our study indicates the potential for an intervention—a remote contraction—that enhances sensory input or motoneuron excitability to increase NMES-evoked torque.

## Supplementary Information

Below is the link to the electronic supplementary material.

Supplementary file1 (DOCX 77 KB)

Supplementary file2 (DOCX 16 KB)

## Data Availability

Data will be made available upon reasonable request.

## Abbreviations

CKC: Convolutive kernel compensation
Δ*F*: Delta frequency
EMG: Electromyographic
EMG_MVC_: Electromyographic activity recorded during a maximal voluntary contraction
MVC: Maximal voluntary contraction
NMES: Neuromuscular electrical stimulation
PIC: Persistent inward currents
RMS: Root mean square
WPHF: Wide-pulse high-frequency
