# Supplementary material for: Motoneuron persistent inward current contribution to increased torque responses to wide-pulse high-frequency neuromuscular electrical stimulation
Source: Eur J Appl Physiol. 2024 Jun 28;124(11):3377–86. doi: 10.1007/s00421-024-05538-8 (PMC11519318; doi:10.1007/s00421-024-05538-8)
Supplement: Supplementary file 1 — Supplementary file1 (DOCX 77 KB) [file 421_2024_5538_MOESM1_ESM.docx]

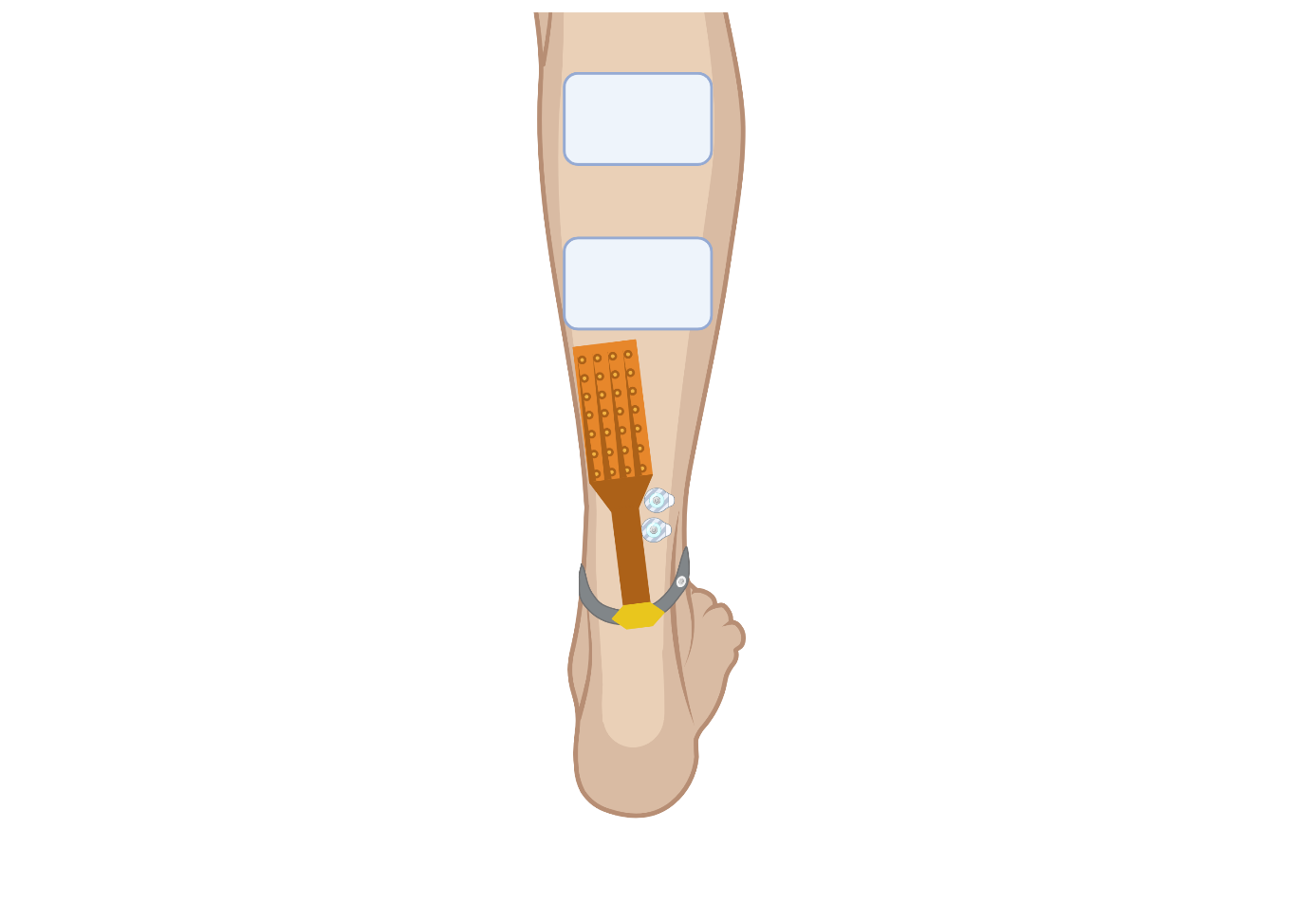


*Supplementary figure 1. Electrode placement in the experimental set-up. Two stimulation electrodes (rectangular, light blue) were positioned on the calf muscle. The high-density electromyographic grid (light brown) and two circular electrodes (bipolar electromyographic recordings, light blue) were positioned on the soleus muscle. The strap electrode around the ankle joint was used as a ground electrode.*
