# Supplementary material for: Motoneuron persistent inward current contribution to increased torque responses to wide-pulse high-frequency neuromuscular electrical stimulation
Source: Eur J Appl Physiol. 2024 Jun 28;124(11):3377–86. doi: 10.1007/s00421-024-05538-8 (PMC11519318; doi:10.1007/s00421-024-05538-8)
Supplement: Supplementary file 2 — Supplementary file2 (DOCX 16 KB) [file 421_2024_5538_MOESM2_ESM.docx]

| *Supplementary Table 1. Number of motor units identified in total, tracked in the three conditions and number of pairs of motor units that were computed for DeltaF calculation.* | | | |
| --- | --- | --- | --- |
| Participant | Number of motor units identified | Number of motor units tracked in all three conditions | Number of pairs of motor units |
| 1 | 17 | 4 | 9 |
| 2 | 15 | 4 | 8 |
| 3 | 24 | 6 | 12 |
| 4 | 25 | 7 | 14 |
| 5 | 17 | 4 | 10 |
| 6 | 15 | 3 | 6 |
| 7 | 6 | 2 | 3 |
| 8 | 0 | 0 | 0 |
| 9 | 0 | 0 | 0 |
| 10 | 29 | 9 | 18 |
|  |  |  |  |
